# The university and the Sustainable Development Goals for achieving the 2030 Agenda: contributions from nursing

**DOI:** 10.1590/0034-7167-2025-0467

**Published:** 2026-09-28

**Authors:** Nair Chase da Silva, Gilsirene Scantelbury de Almeida, Noeli das Neves Toledo, Edson Batista dos Santos

**Affiliations:** I Universidade Federal do Amazonas. Manaus, Amazonas, Brazil

**Keywords:** Sustainable Development, Nursing, Health Education, Universities, Teaching., Desarrollo Sostenible, Enfermería, Educación en Salud, Universidades, Enseñanza.

## Abstract

**Objectives:**

to describe the experience of teaching the Sustainable Development Goals to third-semester nursing students at *Universidade Federal do Amazonas*.

**Methods:**

this is an experience report of teaching the Sustainable Development Goals as content for the “Educational Processes in Health” course from March to July 2025. The course was developed in four stages: theoretical framework presentation; educational project under construction; educational project in motion; and educational project sharing.

**Results:**

academics were introduced to the Sustainable Development Goals and, through collective educational practices, shared the topic with different groups, such as elementary and high school students, outsourced maintenance and cleaning professionals, administrative technicians, and local health council members.

**Conclusions:**

the experience equipped students for a critical and reflective educational practice, incorporating the Sustainable Development Goals into their training.

## RESUMEN

**Objetivos:**

describir la experiencia de enseñanza de los Objetivos de Desarrollo Sostenible a estudiantes de tercer año de enfermería de la *Universidade Federal do Amazonas*.

**Métodos:**

informe de experiencia sobre la enseñanza de los Objetivos de Desarrollo Sostenible como contenido del curso “Procesos Educativos en Salud” de marzo a julio de 2025. El curso se desarrolló en cuatro etapas: presentación del marco teórico; proyecto educativo en construcción; proyecto educativo en marcha; y difusión del proyecto educativo.

**Resultados:**

los estudiantes conocieron los Objetivos de Desarrollo Sostenible y, mediante prácticas educativas colectivas, compartieron el tema con diferentes grupos, como estudiantes de primaria y secundaria, profesionales de mantenimiento y limpieza subcontratados, técnicos administrativos y miembros del consejo local de salud.

**Conclusiones:**

la experiencia capacitó a los estudiantes para una práctica educativa crítica y reflexiva, incorporando los Objetivos de Desarrollo Sostenible en su formación.

## INTRODUCTION

The Sustainable Development Goals (SDGs), aimed at achieving the 2030 Agenda, are an initiative of the United Nations (UN) with the intention that peoples and nations build a better world by reducing social inequalities, protecting the environment, and promoting a just life^(1)^. This movement has been developing since 1972, starting with the Stockholm Conference, which warned about the harmful consequences of environmental degradation. Subsequently, the UN Brundtland Report warned about the outcomes of the uncontrolled use of natural resources and introduced the concept of sustainable development as “development that meets the needs of the present without compromising the ability of future generations to meet their own needs”^(2)^.

Thus, it became a consensus among experts that caring for the planet requires actions and decisions from civil society and governments aligned with economic, social, and environmental sustainability. Consequently, in 1992, the Millennium Development Goals (MDGs) were formulated, comprising Agenda 21, listing eight goals, 21 targets, and 60 indicators focused on global health^(3,4)^.

In September 2015, during the UN General Assembly, 193 member countries decided to create the 2030 Agenda, composed of 17 goals, 169 targets, and 231 indicators. This context allows us to conclude that, when considering global health, the world scenario is complex, given the challenges that arise. Key challenges include filling the gaps not addressed by MDGs, the short timeframe for achieving the goals, and ongoing challenges such as the outbreak of pandemics-as COVID-19-the risk of new pandemics, environmental disasters, and wars. These factors require greater commitment from everyone to address old, new, and future demands^(3,4)^.

Although initiatives have been undertaken, they have achieved little success, such as the creation in 2016 of the Brazilian National Commission for the Sustainable Development Goals (In Portuguese, *Comissão Nacional para os Objetivos de Desenvolvimento Sustentável* - CNODS), which was discontinued the following year. Subsequently, initiatives emerged in higher education institutions (HEIs), with very varied performance among them. More recently, in 2023, the Brazilian commitment was revived with the re-establishment of the CNODS and the adoption of internal initiatives, in an attempt to make up for lost time^(4,5)^. What we are still seeing is a discreet mobilization around the issue, with very specific actions both in academia, the training ground for future professionals, and in healthcare services, where professionals are working, in society in general, and in government spaces.

In most cases, there is a noticeable focus on actions that were already being carried out and that are aligned with the 17 SDG proposals, such as the programmatic actions in health established by the Ministry of Health. However, there is no apparent articulation with the SDG objectives, targets, and indicators at the local level, with a view to achieving better levels globally.

Considering the relevance of the 2030 Agenda, “to eradicate poverty, protect the environment and ensure that people everywhere can enjoy peace and prosperity”, and understanding that HEIs are spaces with the potential to positively impact these purposes through professional training, the *Escola de Enfermagem de Manaus* (EEM) of *Universidade Federal do Amazonas* (UFAM) has been developing, in the last five years, teaching, research, and extension actions identified with the SDGs^(4)^. These actions occur simultaneously with adherence to the Health Promoting Universities (HPUs) movement, whose purposes are convergent.

From this perspective, the SDGs are incorporated into the training of future nurses through teaching in the “Educational Processes in Health” (EPHs) subject. The subject’s structuring axis was the approach to the SDGs in students’ educational practice, considering the interface of nursing with the commitments assumed by 193 countries in favor of the planet.

## OBJECTIVES

To describe the experience of teaching the SDGs to nursing students at UFAM.

## METHODS

This is a descriptive study, structured around an experience report that involved the description of academic experiences related to the teaching-learning process in the “EPHs” subject for the undergraduate nursing course.

The course was offered from March to July 2025, at EEM facilities and in public schools, and involved 33 students under the supervision of three professors, a master’s student in a teaching internship, and an undergraduate student as a course monitor. The course had a workload of 60 hours, 30 of which were theoretical and 30 practical. Content from the fields of education and health was covered. The the course aimed to “equip students with the tools to develop a critical and reflective educational practice, to socialize knowledge, and to contribute to building the autonomy of students and the groups with which they interact”.

This report was not submitted to the Research Ethics Committee, since CNS Resolution 510/2016 waives ethical review for research that exclusively uses public information or reports of professional/academic experiences, without identifying subjects. Nevertheless, the ethical principles of respect, privacy, and integrity of those involved were observed, guaranteeing confidentiality and the non-disclosure of personal or sensitive data.

## RESULTS

### Experience development

The course was developed in four stages. Stage I, entitled “Theoretical framework presentation”, addressed topics related to education, concepts and theories of the teaching-learning process, the potential of education in transforming society, levels of teaching planning, the nurses’ role as a health educator, and the SDGs.

In stage II, entitled “Educational project under construction”, topics related to educational planning, the Brazilian National Curriculum Guidelines for the nursing course, the development of the educational intervention project, and the educational activity plan were discussed.

Stage III, entitled “Educational project in motion”, comprised the development of collective educational practices directed at: elementary, middle, and high school students from the municipal and state education systems; members of the local health council of the basic units that make up the South Health District (In Portuguese, *Distrito de Saúde Sul* - DISASUL); employees of the company providing maintenance and cleaning services to the academic unit; and the technical-administrative staff of EEM.

Finally, in stage IV, called “Educational project socializing”, students shared, in the classroom, through oral and written presentations, the development of the educational practices carried out, reporting experiences, impressions, performance, and knowledge recovered and added.

The course dynamic was action-reflection-action, with students being equipped through specific training sessions on the SDGs. This stage aimed to provide a broad and contextualized understanding of the 2030 Agenda, addressing its origin, structure, goals and indicators, as well as its interface with public health and education policies.

The resources used included reading and discussing key texts from the UN and the Ministry of Health, analyzing thematic videos, discussion groups, and participatory activities that encouraged critical thinking and the practical application of the SDGs in the context of nursing. This prior training allowed students to develop their educational projects in a more conscious and intentional manner, aligning the content with the social, environmental, and economic dimensions of sustainable development, in order to promote confidence and autonomy in conducting activities in different practice settings.

The course was taught in its entirety as presented in the syllabus. It began with mutual introductions between students, professors, the master’s student, and the teaching assistant, followed by a presentation of the group’s expectations. Subsequently, a diagnostic assessment was conducted on the topics selected for the course, and the syllabus was presented. The topics were presented once a week for four hours. Minor adjustments were made to dates and the depth of content, considering the responses from diagnostic assessment.

The teaching and learning strategies adopted with students included reading and discussing selected articles, presenting and discussing videos, sharing statements about experiences in the training process, reviewing the Course Pedagogical Project, gaining preliminary knowledge of the groups and locations selected for educational practices, developing the educational project, discussion groups, gamification, and oral and written presentation of the experience report.

Practical classes were held at the locations where participants were, totaling 33 meetings. The educational activities always emphasized the exchange of knowledge between professors and academics, and between them and their participants, encouraging participants to share the topics within their social and family groups, thus building a supportive, responsible, and empowered network that strengthens and protects everyone.

The topics covered in the primary, secondary, and high school education network were suggested by school management and linked to the SDGs by academics. Among the topics were contraceptive methods, how to administer first aid, violence in schools, respect for differences, children’s and adolescents’ rights, bullying and cyberbullying, healthy eating, promoting personal hygiene, and arboviruses (dengue and chikungunya). For service provider company employees, the topics were autism spectrum disorder and diabetes mellitus. For DISASUL local health council members, the topic was SDGs. For administrative staff, the topics were SDGs for achieving the 2030 Agenda, urban rivers and waterborne diseases, the impacts on public health in the municipality of Manaus, and the 2030 Agenda territorialization in the municipalities of Amazonas.

The activities were carried out in three different basic education schools (high and elementary school) and in a higher education institution (EEM), through four meetings in each of them. Participants included 150 elementary school students, 80 high school students, six staff members, four administrative technicians, and ten DISASUL local health councilors. The teaching strategies adopted were small group discussions, oral presentation of the topic, brainstorming, gamification, group discussions, painting, case studies, storytelling, video presentations, and discussion of selected articles.

The teaching resources used were cards with illustrative figures, posters, whiteboard and markers, projector, computer, poster board, various figures, index cards, cards, and toys. Learning assessment was carried out through gamification techniques, such as the Kahoot platform, preand post-tests, quizzes, question and answer formulation, comments, and case studies. For the collective educational activities, students formed six teams of five to six participants.

After the collective educational practices were completed, students returned to the classroom, where a discussion was held on how to prepare and present oral and written experience reports. This led to the stage called “Educational project sharing”, in which everyone-students and master’s students-had the opportunity to report their experiences both orally and in writing. A post-test was administered, comparing it to the pre-test given at the beginning of the course. Finally, the course was evaluated, reviewing the entire journey of knowledge and experiences accumulated throughout life and incorporating newly acquired knowledge, with the aim of applying them to their educational paths.

From this perspective, the 2030 Agenda was central to the subject, both as a global policy in its macro sense and in each of its 17 objectives, anchoring itself to a greater or lesser extent in its goals and indicators. The choice of the SDGs as the structuring axis of the subject aligned with HEIs’ institutional policy, as well as with EEM’s adherence to the HPU movement, which points to the HEI’s involvement with the university community and its surroundings. It was also considered that the topic is still little known and, when known, not explored in depth. Added to these aspects is the fact that nurses, due to their leadership and professional commitment, are at the forefront of the implementation of the 2030 Agenda, needing to be prepared for it^(6)^.

## REFLECTIONS ON THE LIVED EXPERIENCE

Students’ engagement with educational topics has made them aware of the educational component of nursing practice. Their views on education as an instrument of liberation or domination, theories of teaching and learning, levels of educational planning, and their own training path provide them with this understanding. Although such content needs to be grasped in greater depth, the course has provided access to knowledge that allows them to act based on concepts and theories accepted by the scientific community.

The approach to SDGs broadened their perspectives on sustainable development policies and professionals’ and institutions’ commitment, such as educational institutions, in the pursuit of quality of life, impacting health and vice versa. In this process, the subject enabled the student to acquire new knowledge, expanding their view of the field of health and articulating it with that of education, in addition to navigating local and global health policy, and developing communication and expression skills.

It became evident that information regarding the SDGs has been addressed superficially, being viewed as something worrying. It is understood that, since this is a time when academics are beginning to develop critical thinking and associate it with social issues, their thinking should be more proactive^(7)^. Thus, the valuable implications of introducing this topic for the training of nurses stand out, as it provides a foundation for the development of critical and reflective thinking throughout the academic journey, especially in its articulation with SDGs 3 (Good health and well-being) and 4 (Quality education).

It is reiterated that the university is the ideal space for the dissemination and implementation of the SDGs, given its capacity for scientific and technological production, and its commitment to society, being referred to by some as indispensable for the success of sustainable development^(1)^. A study carried out with professors from basic and higher education considers that, even knowing about the existence of the SDGs, knowledge about them is still superficial and lacks depth regarding their implications and what they entail^(8)^.

The educational practices directed at outsourced company employees were well-received, highlighting the relevance of SDG 3 by promoting access to health information as a strategy for prevention and promotion of quality of life. Demands related to family, community, and neighborhood situations reinforce the importance of health education as an instrument for autonomy, empowerment, and strengthening of care networks, broadening the understanding of sustainability beyond its environmental dimension and incorporating social, educational, and human aspects.

The dialogue established with outsourced company employees was a relevant formative experience for students, especially because it enabled the building of relationships, the exchange of knowledge, and the recognition of knowledge that is often made invisible in the institutional routine. This interaction concretely highlighted principles related to SDG 4 (Quality education), by valuing continuous and inclusive educational processes, as well as to SDG 10 (Reduced inequalities), by challenging social and symbolic hierarchies that permeate educational spaces, and to SDG 16 (Peace, justice and strong institutions), by promoting a culture of peace.

Academics recognized that, despite daily interaction, these individuals were not always perceived as protagonists of knowledge. This implied a significant investment of time and study by academics, favoring the expansion of academic, ethical, and social competencies, fundamental for an education committed to sustainable development. This finding prompted critical reflections on equity, social justice, and the valuing of diverse experiences.

Within the institutional context, the subject contributed to strengthening inter-institutional ties, in line with SDG 17 (Partnerships for the goals), since educational practices in health were developed in teaching units that maintain systematic collaboration with EEM. This articulation enhanced the reach of the actions, reaffirming the university’s role as a promoter of integration between teaching, research, and outreach.

The educational activity for the council members of DISASUL health units provided students with knowledge about how the population participates in the Brazilian Unified Health System (In Portuguese, *Sistema Único de Saúde* - SUS) and gave the council members the opportunity to present the SDGs as a global pact. Thus, third-year students were able to experience both fields of knowledge: health and education, the local and the global. They also learned about a new aspect of nursing practice: health education.

The educational activity for administrative staff on the SDGs provided the group with knowledge of the local/regional health proposal with global reach. This action contributed to strengthening personal bonds and to the EEM’s recognition as a HPU, in line with SDG 4 and SDG 17. By fostering spaces for dialogue and collective learning, the experience reinforced the importance of institutional co-responsibility and integration between different sectors as fundamental strategies for promoting health, sustainability, and strengthening educational practices in the university context.

Hence, it is important to reflect on the presence of SDG 16, which deals with peace, justice, and effective institutions. The subject provided opportunities for good relationships, fostering an environment of harmony and affection, which is conducive to learning^(9)^.

The SDG approach offered in the “EPHs” course brought important gains to the academic training of students, such as an increased demand for training on the subject. However, it was observed that there are still gaps in the knowledge of the subject addressed by students in the institutions involved. This incipient knowledge, identified from discussions about the SDGs, corroborates the literature^(10)^.

Becoming aware that, beyond local and national policies, there is a global policy that seeks to reduce inequalities, promote a culture of peace, reduce hunger, reduce illiteracy, promote gender equality, care for the planet, care for education and health, strengthen institutions, establish partnerships, among other policies, is a way of positioning ourselves and being in the world. This is a space with which nursing, by nature, identifies. Knowing that Brazil achieved most of the MDG targets ahead of schedule and that strategies for information convergence, aligned with the SUS, are being sought for the SDGs, creates conditions to stimulate commitment to improving Brazilian performance, knowing that this will have some global impact^(3,4)^.

Being aware that local policies stem from spurious or solidarity-based international agreements, understanding that we will be affected, sooner or later, by good or bad results, should sensitize everyone to the necessary engagement in the social, environmental, and economic context. The Brundtland Report “Our Common Future” and the 2030 Agenda “leave no one behind” imply that we must be conscious of our attitudes, such as acquiring and sharing knowledge.

### Contributions to nursing, health, and public policy

Sharing knowledge with society is a commitment that all professionals should place on their agenda. By doing so, nursing students learn by teaching, improving their skills and competencies, and applying theory to practice. The study contributed to SDG promotion by integrating the 2030 Agenda into nursing education, strengthening students’ critical and civic education. The educational actions favored health promotion, quality education, and inequality reduction. Furthermore, they encouraged inter-institutional partnerships and community engagement in favor of sustainable development.

## FINAL CONSIDERATIONS

The experience report on teaching the SDGs as content in the “EPHs” subject achieved its objective. Students gained knowledge about the SDGs as a global pact to which Brazil is a signatory, and were equipped to develop a critical and reflective educational practice. They were encouraged to share knowledge and contribute to building autonomy, both their own and that of the groups with which they interacted.

The implications of this experience for the disciplinary field and for advances in knowledge and practice stand out, such as the inclusion of students in discussions relevant to sustainable development, bringing them closer to different actors in society (administration professionals and health agents) and basic education schools. Such discussions will contribute to the training of both professionals and critical and reflective citizens about the context in which they live and the achievement of the goals for 2030.

The experience exceeded academics’ expectations by adding new elements to their training, such as building relationships with workers who share the same physical space that were previously “non-existent”, discovering the pleasure of sharing knowledge, and developing their identity as nurses.

## Data Availability

The research data are available within the article.
