# Supplementary material for: The university and the Sustainable Development Goals for achieving the 2030 Agenda: contributions from nursing
Source: Rev Bras Enferm. 2026 Sep 28;79:e20250467. doi: 10.1590/0034-7167-2025-0467 (PMC13623386; doi:10.1590/0034-7167-2025-0467)
Supplement: Translation

# Translation: Universidade e os Objetivos de Desenvolvimento Sustentável para alcance da Agenda 2030: contribuições da enfermagem

**DOI:** 10.1590/0034-7167-2025-0467pt

**Authors:** Nair Chase da Silva, Gilsirene Scantelbury de Almeida, Noeli das Neves Toledo, Edson Batista dos Santos

**Affiliations:** I Universidade Federal do Amazonas. Manaus, Amazonas, Brasil

**Keywords:** Desenvolvimento Sustentável, Enfermagem, Educação em Saúde, Universidades, Ensino.

## Abstract

**Objetivos:**

descrever a experiência vivenciada no ensino dos Objetivos de Desenvolvimento Sustentável para acadêmicos do terceiro período do curso de enfermagem da Universidade Federal do Amazonas.

**Métodos:**

relato de experiência do ensino dos Objetivos de Desenvolvimento Sustentável, como conteúdo da disciplina “Processos Educacionais em Saúde”, no período de março a julho de 2025. O desenvolvimento da disciplina ocorreu em quatro etapas: apresentação do suporte teórico; projeto educativo em construção; projeto educativo em movimento; e socializando o projeto educativo.

**Resultados:**

os acadêmicos tiveram contato com os Objetivos de Desenvolvimento Sustentável, e, por meio de práticas educativas coletivas, compartilharam o tema com os diferentes grupos, como estudantes do ensino fundamental e médio, profissionais terceirizados de conservação e limpeza, técnicos administrativos, e membros do conselho local de saúde.

**Conclusões:**

a experiência instrumentalizou os acadêmicos para uma prática educativa crítica e reflexiva, incorporando os Objetivos de Desenvolvimento Sustentável à sua formação.

## INTRODUÇÃO

Os Objetivos de Desenvolvimento Sustentável (ODS), voltados ao alcance da Agenda 2030, constituem uma iniciativa da Organização das Nações Unidas (ONU) com a intenção de que os povos e as nações construam um mundo melhor por meio da diminuição das desigualdades sociais, da proteção do meio ambiente e da promoção de uma vida justa^(1)^. Esse movimento vem sendo desenvolvido desde 1972, a partir da Conferência de Estocolmo, que alertou sobre as consequências nefastas da degradação ambiental. Em seguida, o Relatório Brundtland da ONU advertiu sobre os desfechos do uso descontrolado dos recursos naturais e introduziu a concepção de desenvolvimento sustentável como sendo “aquele que atende às necessidades do presente sem comprometer a possibilidade de as gerações futuras atenderem às suas próprias necessidades”^(2)^.

Assim, tornou-se consenso entre os especialistas que o cuidado com o planeta exige ações e decisões da sociedade civil e dos governantes alinhadas à sustentabilidade econômica, social e ambiental. Consequentemente, em 1992, foram formulados os Objetivos de Desenvolvimento do Milênio (ODM), que compõem a Agenda 21, elencando oito objetivos, 21 metas e 60 indicadores voltados à saúde global^(3,4)^.

Em setembro de 2015, durante a Assembleia Geral da ONU, 193 países-membros decidiram pela criação da Agenda 2030, composta por 17 objetivos, 169 metas e 231 indicadores. Esse contexto permite concluir que, ao se pensar em saúde global, o cenário mundial é complexo, diante dos desafios que se apresentam. Destacam-se o preenchimento das lacunas não atendidas pelos ODM, o curto espaço de tempo para o alcance das metas e os desafios em curso, como a eclosão de pandemias - a exemplo da COVID-19 -, o risco de novas pandemias, os desastres ambientais e as guerras. Esses fatores requerem maior adesão de todos para enfrentar as velhas, novas e futuras demandas^(3,4)^.

No Brasil, ainda que iniciativas tenham sido tomadas, lograram pouco êxito, a exemplo da criação, em 2016, da Comissão Nacional para os Objetivos de Desenvolvimento Sustentável (CNODS), que foi descontinuada no ano seguinte. Posteriormente, surgiram iniciativas nas instituições de ensino superior (IESs), com desempenho muito variado entre elas. Mais recentemente, em 2023, o compromisso brasileiro foi resgatado com a recriação da CNODS e a adoção de iniciativas internas, na tentativa de recuperar o tempo perdido^(4,5)^. O que ainda se observa é uma discreta mobilização em torno do assunto, com ações bastante pontuais tanto na academia, espaço de formação dos futuros profissionais, quanto nos serviços de saúde, onde os profissionais estão trabalhando, na sociedade em geral e nos espaços de governo.

Na maioria dos casos, percebe-se uma atuação sobre as ações que já vinham sendo realizadas e que são convergentes com as propostas dos 17 ODS, a exemplo das ações programáticas em saúde estabelecidas pelo Ministério da Saúde. Contudo, não se observa uma articulação aparente com os objetivos, metas e indicadores dos ODS, a nível local, na perspectiva de alcançar melhores patamares no âmbito global.

Considerando a relevância da Agenda 2030, “erradicar a pobreza, proteger o meio ambiente e garantir que, em todos os lugares, as pessoas possam desfrutar de paz e prosperidade”, e entendendo serem as IESs espaços com potencial para impactar positivamente esses propósitos pela via da formação profissional, a Escola de Enfermagem de Manaus (EEM) da Universidade Federal do Amazonas (UFAM) vem desenvolvendo, nos últimos cinco anos, ações de ensino, pesquisa e extensão identificadas com os ODS^(4)^. Essas ações ocorrem simultaneamente à adesão ao movimento das Universidades Promotoras de Saúde (UPSs), cujos propósitos são convergentes.

Nessa perspectiva, inserem-se os ODS na formação dos futuros enfermeiros pela via do ensino na disciplina “Processos Educacionais em Saúde” (PESs). A disciplina teve como eixo estruturante a abordagem dos ODS na prática educativa dos acadêmicos, considerando a interface da enfermagem com os compromissos assumidos por 193 países em favor do planeta.

## OBJETIVOS

Descrever a experiência vivenciada no ensino dos ODS para acadêmicos do curso de enfermagem da UFAM.

## MÉTODOS

Trata-se de estudo descritivo, estruturado a partir de relato de experiência que envolveu a descrição de vivências acadêmicas relacionadas ao processo de ensino-aprendizagem na disciplina “PESs” para o curso de graduação em enfermagem.

A oferta da disciplina ocorreu no período de março a julho de 2025, nas dependências da EEM e em escolas públicas, e contou com 33 acadêmicos sob supervisão de três docentes, uma mestranda em estágio de docência e uma graduanda como monitora da disciplina. A carga horária da disciplina foi de 60 horas, sendo 30 teóricas e 30 práticas. Foram abordados conteúdos dos campos da educação e da saúde. O objetivo da disciplina era de “instrumentalizar o acadêmico para o desenvolvimento de uma prática educativa crítica e reflexiva, socializar conhecimentos e contribuir para a construção da autonomia dos estudantes e dos grupos com os quais interagem”.

O presente relato não foi submetido ao Comitê de Ética em Pesquisa, uma vez que a Resolução CNS nº 510/2016 dispensa apreciação ética em pesquisas que utilizam exclusivamente informações públicas ou relatos de experiências profissionais/acadêmicas, sem identificação de sujeitos. Ainda assim, foram observados os princípios éticos do respeito, da privacidade e da integridade dos envolvidos, garantindo a confidencialidade e a não exposição de dados pessoais ou sensíveis.

## RESULTADOS

### Desenvolvimento da experiência

O desenvolvimento da disciplina ocorreu em quatro etapas. A etapa I, denominada “Apresentação do suporte teórico”, abordou temas relacionados à educação, às concepções e teorias do processo de ensino-aprendizagem, ao potencial da educação na transformação da sociedade, aos níveis de planejamento de ensino, ao papel do enfermeiro como educador em saúde e aos ODS.

Na etapa II, intitulada “O projeto educativo em construção”, foram discutidos temas referentes ao planejamento educacional, às Diretrizes Curriculares Nacionais para o curso de enfermagem, à elaboração do projeto de intervenção educativa e ao plano de atividade educativa.

A etapa III, denominada “O projeto educativo em movimento”, compreendeu o desenvolvimento de práticas educativas coletivas dirigidas: aos estudantes do ensino básico, fundamental e médio das redes municipal e estadual de ensino; aos membros do conselho local de saúde das unidades básicas que compõem o Distrito de Saúde Sul (DISASUL); aos funcionários da empresa prestadora de serviços de manutenção e limpeza da unidade acadêmica; e aos servidores técnico-administrativos da EEM.

Por fim, na etapa IV, denominada “Socializando o projeto educativo”, os acadêmicos compartilharam, em sala de aula, por meio de apresentações orais e escritas, o desenvolvimento das práticas educativas realizadas, relatando vivências, impressões, desempenho e saberes resgatados e agregados.

A dinâmica da disciplina foi de ação-reflexão-ação, sendo os acadêmicos instrumentalizados por meio de encontros formativos específicos sobre os ODS. Essa etapa teve como propósito proporcionar uma compreensão ampla e contextualizada acerca da Agenda 2030, abordando sua origem, estrutura, metas e indicadores, bem como sua interface com as políticas públicas de saúde e educação.

Os recursos utilizados foram leitura e discussão de textos-base da ONU e do Ministério da Saúde, análise de vídeos temáticos, rodas de conversa e dinâmicas participativas que estimularam o pensamento crítico e a aplicação prática dos ODS no contexto da enfermagem. Essa formação prévia permitiu que os acadêmicos elaborassem seus projetos educativos de forma mais consciente e intencional, alinhando os conteúdos às dimensões social, ambiental e econômica do desenvolvimento sustentável, de modo a favorecer a segurança e a autonomia na condução das atividades nos diferentes cenários de prática.

A disciplina foi ministrada integralmente conforme apresentada no plano de ensino. Iniciou-se com a apresentação mútua entre os acadêmicos, professores, a mestranda e a monitora, seguida da apresentação das expectativas do grupo. Em seguida, foi realizada a avaliação diagnóstica sobre os temas selecionados para compor a disciplina, e foi apresentado o plano de ensino desta. Os temas foram apresentados uma vez por semana, com duração de quatro horas. Foram feitos pequenos ajustes de datas e de aprofundamento de conteúdos, considerando as respostas da avaliação diagnóstica.

As estratégias de ensino-aprendizagem adotadas com os acadêmicos foram leitura e discussão de artigos selecionados, apresentação e discussão de vídeos, depoimentos sobre vivências no processo de formação, exame do Projeto Pedagógico de Curso, conhecimento preliminar dos grupos e locais selecionados para as práticas educativas, construção do projeto educativo, rodas de conversa, gamificação, apresentação oral e escrita do relato de experiência.

As aulas práticas foram realizadas nos locais onde se encontravam os interlocutores, perfazendo um total de 33 encontros. As atividades educativas sempre enfatizaram a troca de conhecimentos entre professores e acadêmicos, e entre estes e seus interlocutores, com estímulo para que os interlocutores compartilhassem os temas em seus grupos sociais e familiares, construindo assim uma rede solidária, responsável e empoderada, que fortalece e protege a todos.

Os temas abordados na rede de ensino básico, fundamental e médio foram sugeridos pela gestão escolar e associados aos ODS pelos acadêmicos. Entre os temas estão métodos contraceptivos, como prestar os primeiros socorros, violência na escola, respeito às diferenças, direitos das crianças e dos adolescentes, *bullying* e *cyberbullying*, alimentação saudável, promoção da higiene pessoal, e arboviroses (dengue e chikungunya). Para os funcionários da empresa prestadora de serviço, os temas foram transtorno do espectro autista e diabetes mellitus. Para os membros do conselho local de saúde do DISASUL, o tema foi ODS. Para os técnicos administrativos, os temas foram ODS para o alcance da Agenda 2030, rios urbanos e doenças de veiculação hídrica, os impactos na saúde pública no município de Manaus, e a territorialização da Agenda 2030 nos municípios do Amazonas.

As atividades foram realizadas em três escolas de ensino básico distintas (ensino médio e fundamental) e em escola de ensino superior (EEM), por meio de quatro encontros em cada uma delas. Participaram das atividades 150 estudantes do ensino fundamental, 80 do ensino médio, seis colaboradores, quatro técnicos administrativos e dez conselheiros locais de saúde do DISASUL. As estratégias de ensino adotadas foram discussão em pequenos grupos, exposição oral do tema, tempestade de ideias, gamificação, rodas de conversa, pintura, estudo de caso, *storytelling,* apresentação de vídeos e discussão de artigos selecionados.

Os recursos de ensino utilizados foram cartões com figuras ilustrativas, cartazes, quadro branco e pincéis, *datashow*, computador, cartolinas, figuras diversas, tarjetas, *cards* e brinquedos. A avaliação da aprendizagem ocorreu por meio de técnicas de gamificação, como a plataforma Kahoot, prée pós-testes, quizzes, formulação de perguntas e respostas, comentários, e estudo de caso. Para a realização das atividades educativas coletivas, os acadêmicos compuseram seis equipes de cinco a seis participantes.

Concluídas as práticas educativas coletivas, os acadêmicos retornaram à sala de aula, sendo discutido como elaborar e apresentar relato de experiência oral e escrito. Deu-se, assim, a etapa denominada “Compartilhando o projeto educativo”, na qual todos, acadêmicos e mestranda, tiveram a oportunidade de relatar, tanto oralmente quanto por escrito, suas experiências. Foi aplicado pós-teste, cotejando-se com o pré-teste aplicado no início da disciplina. Por fim, foi realizada a avaliação da disciplina, resgatando todo o percurso dos saberes e das experiências acumuladas ao longo da vida e agregando as recém-adquiridas, na perspectiva de aplicá-las em seus itinerários formativos.

Nessa perspectiva, o tema Agenda 2030 foi central à disciplina enfocada, tanto como uma política global, em seu sentido macro, quanto em cada um de seus 17 objetivos, ancorando-se em maior ou menor profundidade em suas metas e indicadores. A escolha do tema dos ODS para alcance da Agenda como eixo estruturante da disciplina alinhou-se à política institucional da IES, assim como à aderência da EEM ao movimento da UPS, que aponta para uma atuação da IES junto à comunidade universitária e seu entorno. Também se considerou que o tema ainda é pouco conhecido e quando conhecido pouco aprofundado. Agrega-se a esses aspectos o fato de os enfermeiros, devido à sua liderança e ao compromisso profissional, estarem na linha de frente da realização da Agenda 2030, necessitando estar preparado para tal^(6)^.

## REFLEXÕES SOBRE A EXPERIÊNCIA VIVENCIADA

O contato dos acadêmicos com temas da área da educação lhes fez perceber o componente educativo da prática do enfermeiro. A visão sobre a educação como instrumento de libertação ou dominação, as teorias de ensino e aprendizagem, os níveis de planejamento educacional e o itinerário de sua própria formação lhes dão essa possibilidade. Ainda que tais conteúdos precisem ser apreendidos com maior profundidade, a disciplina oportunizou o acesso a saberes que lhes permitem atuar, fundamentados em conceitos e teorias aceitas pela comunidade científica.

A abordagem sobre ODS ampliou seus olhares sobre as políticas de desenvolvimento sustentável e o compromisso do profissional e das instituições, como as de ensino, na busca pela qualidade de vida, impactando a saúde e vice-versa. Nesse movimento, a disciplina possibilitou ao acadêmico adquirir novos conhecimentos, ampliando seu olhar sobre o campo da saúde e articulando-o com o da educação, além de transitar na política de saúde local e global, e desenvolver a capacidade de comunicação e expressão.

Evidenciou-se que as informações acerca dos ODS vêm sendo trabalhadas de forma superficial, sendo vistas como algo preocupante. Entende-se que, por se tratar de um momento em que os acadêmicos iniciam a formação e o desenvolvimento do pensamento crítico, associando-o a questões de ordem social, ele deve ser mais propositivo^(7)^. Assim, Destacam-se as valiosas implicações que a introdução desse tema tem na formação de enfermeiros, pois fornece substrato para o desenvolvimento do pensamento crítico e reflexivo ao longo do percurso acadêmico, sobretudo em sua articulação com os ODS 3 (Saúde e bem-estar) e 4 (Educação de qualidade).

Reitera-se que a universidade é espaço por excelência para difusão e implementação dos ODS, haja vista sua capacidade de produção científica e tecnológica, e seu compromisso com a sociedade, sendo referido por alguns como indispensável para o êxito do desenvolvimento sustentável^(1)^. Estudo realizado junto a docentes do ensino básico e superior considera que, mesmo sabendo sobre a existência dos ODS, o conhecimento sobre eles ainda é superficial e pouco aprofundado quanto às suas implicações e ao que carrega em seu entorno^(8)^.

As práticas educativas direcionadas aos funcionários da empresa terceirizada apresentaram boa aceitação, evidenciando a pertinência do ODS 3, ao promover o acesso à informação em saúde como estratégia de prevenção e promoção da qualidade de vida. As demandas relacionadas a situações familiares, comunitárias e de vizinhança reforçam a importância da educação em saúde como instrumento de autonomia, empoderamento e fortalecimento das redes de cuidado, ampliando a compreensão de sustentabilidade para além de sua dimensão ambiental e incorporando aspectos sociais, educacionais e humanos.

O diálogo estabelecido com os funcionários da empresa terceirizada foi uma experiência formativa relevante para os acadêmicos, sobretudo por possibilitar a construção de vínculos, a troca de saberes e o reconhecimento de conhecimentos frequentemente invisibilizados no cotidiano institucional. Tal interação evidenciou, de maneira concreta, princípios relacionados ao ODS 4 (Educação de qualidade), ao valorizar processos educativos contínuos e inclusivos, bem como ao ODS 10 (Redução das desigualdades), ao tensionar hierarquias sociais e simbólicas que atravessam os espaços educacionais, e ao ODS 16 (Paz, justiça e instituições eficazes), ao promover a cultura da paz.

Os acadêmicos reconheceram que, apesar do convívio diário, esses sujeitos nem sempre eram percebidos como protagonistas de saberes. Isso implicou um significativo investimento por parte dos acadêmicos de tempo e estudo, favorecendo a ampliação de competências acadêmicas, éticas e sociais, fundamentais para uma formação comprometida com o desenvolvimento sustentável. Tal constatação suscitou reflexões críticas acerca da equidade, da justiça social e da valorização da diversidade de experiências.

No âmbito institucional, a disciplina contribuiu para o fortalecimento de vínculos interinstitucionais, em consonância com o ODS 17 (Parcerias e meios de implementação), uma vez que as práticas educativas em saúde foram desenvolvidas em unidades de ensino que mantêm colaboração sistemática com a EEM. Essa articulação potencializou o alcance das ações, reafirmando o papel da universidade como promotora de integração entre ensino, pesquisa e extensão.

A atividade educativa para os conselheiros das unidades de saúde do DISASUL oportunizou aos acadêmicos o conhecimento sobre como se dá a participação da população no Sistema Único de Saúde (SUS) e, aos conselheiros, a oportunidade de apresentar os ODS como um pacto global. Assim, acadêmicos do terceiro período puderam vivenciar os dois campos do saber: a saúde e a educação, o local e o global. Eles também conheceram um novo aspecto da prática do enfermeiro: a educação em saúde.

A atividade educativa para os técnicos administrativos sobre os ODS oportunizou ao grupo o conhecimento da proposta de saúde local/regional com alcance global. Essa ação contribuiu para o estreitamento de vínculos pessoais e para o reconhecimento da EEM como uma UPS, em consonância com o ODS 4 e o ODS 17. Ao fomentar espaços de diálogo e aprendizagem coletiva, a experiência reforçou a importância da corresponsabilização institucional e da integração entre diferentes setores como estratégias fundamentais para a promoção da saúde, da sustentabilidade e do fortalecimento das práticas educativas no contexto universitário.

Nesse sentido, é importante refletir sobre a presença do ODS 16, que trata da paz, justiça e instituições eficazes. A disciplina oportunizou as boas relações, favorecendo um ambiente de harmonia e de afetividade, o que é favorável à aprendizagem^(9)^.

A abordagem dos ODS oferecida na disciplina “PESs” trouxe ganhos importantes à formação dos acadêmicos, como o aumento da demanda de formação sobre o tema. No entanto, pôde-se observar que ainda há lacunas quanto ao conhecimento do tema abordado pelos acadêmicos nas instituições envolvidas. Essa incipiência, identificada a partir das discussões acerca dos ODS, corrobora a literatura^(10)^.

Tomar conhecimento de que, para além das políticas locais e nacionais, existe uma política global que busca diminuir desigualdades, promover a cultura da paz, reduzir a fome, reduzir o analfabetismo, promover a igualdade de gênero, cuidar do planeta, cuidar da educação e da saúde, fortalecer instituições, estabelecer parcerias, entre outras políticas, é uma forma de nos colocarmos e de estarmos no mundo. Esse é um espaço com o qual a enfermagem, por natureza, se identifica. Saber que o Brasil alcançou a maioria das metas dos ODM antes do prazo previsto e que estratégias para a convergência de informações, alinhadas ao SUS, estão sendo buscadas para os ODS, cria condições para estimular o compromisso em melhorar o desempenho brasileiro, sabendo que isso causará algum impacto global^(3,4)^.

Ter consciência também de que políticas locais são provenientes de acordos internacionais espúrios ou solidários, entendendo que seremos atingidos, mais cedo ou mais tarde, por resultados ruins ou bons, deve sensibilizar a todos para o engajamento necessário no contexto social, ambiental e econômico. O “Nosso Futuro Comum” do Relatório Brundtland e o “não deixar ninguém para trás” da Agenda 2030 implicam termos consciência sobre as nossas atitudes, como a de adquirir e compartilhar saberes.

### Contribuições para a área da enfermagem, saúde e política pública

Compartilhar conhecimento com a sociedade é um compromisso que todos os profissionais deveriam colocar na sua agenda. Ao fazê-lo, o enfermeiro em formação aprende ensinando, aperfeiçoando as suas habilidades e competências, e aplicando a teoria à prática. O estudo contribuiu para a promoção dos ODS, ao integrar a Agenda 2030 ao ensino em enfermagem, fortalecendo a formação crítica e cidadã dos acadêmicos. As ações educativas favoreceram a promoção da saúde, a educação de qualidade e a redução das desigualdades. Além disso, estimularam parcerias interinstitucionais e o engajamento comunitário em prol do desenvolvimento sustentável.

## CONSIDERAÇÕES FINAIS

O relato de experiência do ensino dos ODS como conteúdo da disciplina “PESs” teve seu objetivo alcançado. Os acadêmicos agregaram conhecimentos sobre os ODS como um pacto global do qual o Brasil é signatário, e foram instrumentalizados para o desenvolvimento de uma prática educativa crítica e reflexiva. Eles foram estimulados a socializar conhecimentos e a contribuir para a construção da autonomia, tanto a deles próprios quanto a dos grupos com os quais interagiram.

Destacam-se as implicações dessa experiência para o campo disciplinar e para os avanços no conhecimento e na prática, tais como inserção dos discentes em discussões relevantes para o desenvolvimento sustentável, aproximando-os aos diferentes atores da sociedade (profissionais da administração e agentes da saúde) e escolas de ensino básico. Tais discussões contribuirão para formação tanto de profissionais quanto de cidadãos críticos e reflexivos sobre o contexto em que vivem e o alcance das metas para 2030.

A experiência extrapolou a expectativa dos acadêmicos ao agregar novos elementos à sua formação, como a construção de vínculos com os trabalhadores que convivem no mesmo espaço físico até então “inexistentes”, a descoberta do prazer de compartilhar saberes e a construção do ser enfermeiro.

## Data Availability

Os dados de pesquisa estão disponíveis no corpo do artigo.
